# First cytogenetic information for five Nilotic elephantfishes and a problem of ancestral karyotype of the family Mormyridae (Osteoglossiformes)

**DOI:** 10.3897/CompCytogen.14i3.52727

**Published:** 2020-08-19

**Authors:** Sergey Simanovsky, Dmitry Medvedev, Fekadu Tefera, Alexander Golubtsov

**Affiliations:** 1 Severtsov Institute of Ecology and Evolution, Russian Academy of Sciences, 33 Leninskij prosp., Moscow, 119071, Russia Russian Academy of Sciences Moscow Russia; 2 National Fishery and Aquatic Life Research Center, Ethiopian Institute of Agricultural Research, Sebeta, P.O. Box 64, Ethiopia Ethiopian Institute of Agricultural Research Sebeta Ethiopia

**Keywords:** Africa, chromosomes, karyotype evolution, *
Brevimyrus
*, *
Cyphomyrus
*, *
Hippopotamyrus
*, *
Marcusenius
*, *
Mormyrops
*

## Abstract

The elephantfish family Mormyridae is the most diverse lineage of the primitive teleostean clade Osteoglossomorpha distributed in inland waters of all continents except Antarctica and Europe. The family Mormyridae is endemic to Africa and includes 22 genera and almost 230 species. The evolutionary radiation of mormyrids most probably should be attributed to their capability of both generating and receiving weak electric signals. Up-to-date cytogenetic studies have revealed substantial karyotype differentiation among the nine investigated elephantfish species and genera (a single species studied per each genus). In the present study, karyotypes of five species representing five mormyrid genera (four unexplored ones) collected from the White Nile system in southwestern Ethiopia are described for the first time. The results show substantial variety of the diploid chromosome and fundamental numbers: 2n = 48 and FN = 54 in *Brevimyrus niger* (Günther, 1866), 2n = 50 and FN = 72 in *Cyphomyrus petherici* (Boulenger, 1898), 2n = 50 and FN = 78 in *Hippopotamyrus pictus* (Marcusen, 1864), 2n = 50 and FN = 76 in *Marcusenius cyprinoides* (Linnaeus, 1758), 2n = 52 and FN = 52 in *Mormyrops anguilloides* (Linnaeus, 1758). Karyotype structure in the latter species seems to be close to the ancestral condition for the family. This hypothesis is discussed in the light of available data on karyotype diversity and phylogeny of mormyrids.

## Introduction

The elephantfish family Mormyridae belongs to one of the most primitive groups of teleostean fishes, the cohort Osteoglossomorpha (Nelson et al. 2016). The family is endemic to the African continent and includes 22 genera and almost 230 species (Froese and Pauly 2019; Eschmeyer et al. 2020). In genus and species diversity it exceeds all other extant osteoglossomorph lineages. The evolutionary radiation of mormyrids most probably should be attributed to their ability of both generating and receiving weak electric signals that provides dual functions of ‘electrolocation’ and communication (Hopkins 2009, Carlson and Arnegard 2011).

First cytogenetic data on the osteoglossomorphs and particularly mormyrids were published by Hinegardner and Rosen (1972) and Uyeno (1973) almost half a century ago. Thereafter, the karyotype structure and cellular DNA content of osteoglossomorphs were progressively studied (reviewed by Arai 2011; Canitz et al. 2016; Barby et al. 2018; Cioffi et al. 2019). The recent works on mormyrids (Krysanov and Golubtsov 2014; Ozouf-Costaz et al. 2015; Canitz et al. 2016) raised to nine the number of mormyrid genera studied. The number of species studied is also nine because one species only has been karyotyped for all genera. The diploid chromosome numbers in most mormyrids are similar (2n = 48 or 50 excepting *Pollimyrus* Taverne, 1971 with 2n = 40). Nevertheless, the varying bi-armed chromosome numbers and ‘amazing’ diversity in NOR positions and C-banding patterns provide evidence for the substantial divergence in the karyotype structure with the dominating role of pericentric inversions (Ozouf-Costaz et al. 2015).

There is a coherent hypothesis about phylogenetic position of the family Mormyridae among other Osteoglossomorpha (Lavoué and Sullivan 2004; Inoue et al. 2009; Nelson et al. 2016). The phylogenetic structure of mormyrids themselves is not well-elaborated, but three basal groups in their radiation (the genera *Petrocephalus* Marcusen, 1854; *Myomyrus* Boulenger, 1898; *Mormyrops* Müller, 1843) are reliably defined (Alves-Gomes and Hopkins 1997; Sullivan et al. 2000; Lavoué et al. 2003). This makes it possible to hypothesize about the mormyrid karyotype evolution. Based on available data Canitz et al. (2016) suggested for Mormyridae the ancestral chromosome number 2n = 48–50, that is well-coordinated with the hypothetical ancestral karyotype for the teleostean fishes and early vertebrates in general (Ohno et al. 1969; Jaillon et al. 2004; Kohn et al. 2006; Nakatani et al. 2007).

Meanwhile, only a small fraction of the total mormyrid diversity (less than 5% of species) has been yet studied cytogenetically. New findings may correct the existing views on their karyotype evolution. In the present study, new data for five mormyrid species from northern East Africa are presented using cytogenetic analysis (chromosome number and morphology). Relevance of these data to undrstanding of karyotype evolution within the family Mormyridae is considered.

## Material and methods

The fifteen individuals studied represent five species of different genera – *Brevimyrus niger* (Günther, 1866), *Cyphomyrus petherici* (Boulenger, 1898), *Hippopotamyrus pictus* (Marcusen, 1864), *Marcusenius cyprinoides* (Linnaeus, 1758) and *Mormyrops anguilloides* (Linnaeus, 1758) – of the elephantfish family Mormyridae (Table 1). Fish were collected in southwestern Ethiopia under the umbrella of the Joint Ethiopian-Russian Biological Expedition (JERBE) at three sites in November of 2017: the Baro River downstream of the City of Itang (8°10'47"N, 34°15'2"E), the Tida River half way between the cities of Gambela and Itang (8°16'15"N, 34°25'52"E) and the Alvero River downstream of the Abobo Dam (7°52'23"N, 34°29'48"E). All three rivers belong to the Sobat River drainage discharging into the White Nile in South Sudan. Fish were caught with cast or gill nets, delivered in 80-l plastic containers into the field laboratory, where they were kept in permamently aerated water for several hours before treatment.

**Table 1.** Species, fish standard length (SL), numbers of individuals (N) and metaphases (N_mt_) studied, and collection site.

Species	SL, mm	N	N_mt_	Collection site
*Brevimyrus niger*	81–87	3 (1♀, 2♂)	32	Tida River
*Cyphomyrus petherici*	69–153	5 (3♀, 2♂)	54	Alvero River
*Hippopotamyrus pictus*	197	1 (♂)	11	
*Marcusenius cyprinoides*	196–217	3 (2♀, 1♂)	30	
*Mormyrops anguilloides*	409–498	2 (1♀, 1♂)	21	
	413	1 (♀)	17	Baro River

Before preparation fish were treated intraperitoneally with 0.1% colchicine for 3–4 hours. Then fish were euthanized with an overdose of tricaine methanesulfonate (MS-222), identified based on morphological key characters (Golubtsov et al. 1995, Levin and Golubtsov 2018), measured to an accuracy of 1 mm, dissected for gonad examination and tissue sampling, and preserved in 10% formaldehyde. Vouchers are deposited at the Institute of Ecology and Evolution (Moscow) under provisional labels of JERBE.

Chromosome preparations were obtained from anterior kidney according to Kligerman and Bloom (1977). Briefly, the anterior kidney tissue was incubated with 0.075M KCl hypotonic solution for 20–30 min at room temperature and fixed with 3:1 methanol : acetic acid. To prepare slides a fixed tissue was incubated with 50% glacial acetic acid, suspended, and dropped onto a hot slides. Air-dried chromosome spreads were stained conventionally with 4% Giemsa solution in phosphate buffer at pH 6.8 for 8 min.

Chromosome spreads were analysed under “Axioplan 2 Imaging” microscope (Carl Zeiss, Germany) equipped with “CV-M4+CL” camera (JAI, Japan) and “Ikaros” software (MetaSystems, Germany). Karyotypes were established according to the centromere position following the nomenclature of Levan et al. (1964). Chromosomes were classified as metacentric (a), submetacentric (sm) and acrocentric (a), including subtelocentric and telocentric chromosomes, and grouped according to their morphology in order of decreasing size. To determine the fundamental number (FN), metacentrics and submetacentrics were considered bi-armed and acrocentrics as uni-armed. The number of complete metaphase plates studied for each specimen is presented in Table 1.

## Results and discussion

**Figure 1.**
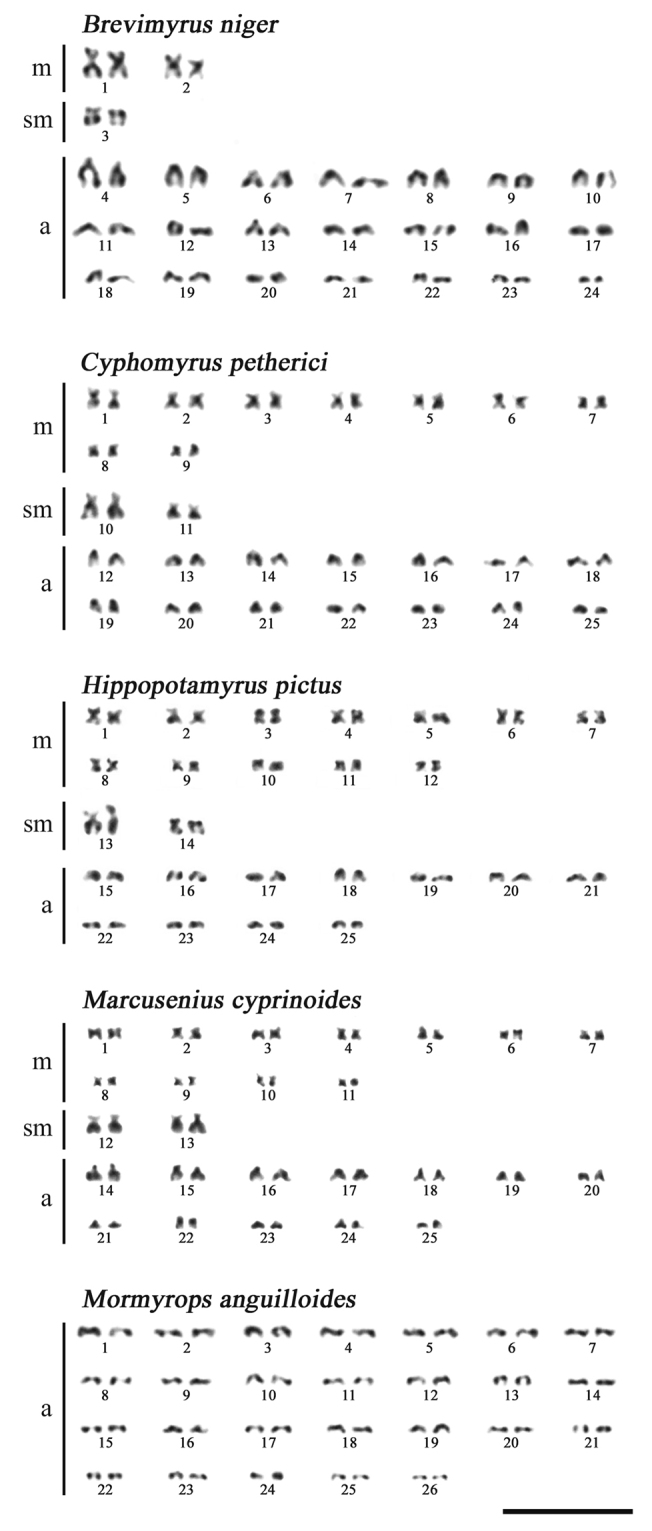
Karyotypes of five elephantfishes of the family Mormyridae. Scale bar: 10 μm.

*Brevimyrus niger* has a karyotype with 2n = 48 (Fig. 1) consisting of 4 metacentrics (m), 2 submetacentrics (sm) and 42 acrocentrics (a). Three taxa share the same diploid numbers of chromosomes 2n = 50 but differ in karyotypic formula: *Cyphomyrus petherici* has 18m, 4sm and 28a, *Hippopotamyrus pictus* has 24m, 4sm and 22a, and *Marcusenius cyprinoides* has 22m, 4sm and 24a. Finally, *Mormyrops anguilloides* has karyotype with 2n = 52 consisting exclusively of acrocentrics gradually decreasing in size. In the other species studied by us one or two pairs of metacentrics or submetacentrics noticeably exceed in size most acrocentrics that admits an origin of the larger chromosomes via the centric fusions.

No distinguishable sex chromosomes were observed in complements of the four species in which individuals of both sexes were studied (*B. niger*, *C. petherici*, *M. cyprinoides*, and *M. anguilloides*), while the only male of *H. pictus* was karyotyped (Table 1). This is in agreement with the lack of reports on sex chromosomes in other mormyrids, but presence of heteromorphic sex chromosomes was supposed in the Asian arowana *Scleropages formosus* (Müller & Schlegel, 1840) from the family Osteoglossidae distantly related to Mormyridae (Bian et al. 2016; but see Cioffi et al. 2019).

Data for all mormyrid taxa studied cytogenetically in the present study and earlier are presented in Table 2. Taxa within the subfamily Mormyrinae are listed in alphabetical order. Recognition of the subfamily Petrocephalinae, as a sister group to all other mormyrids, is well-grounded by morphological (including structure of electrocytes) and molecular phylogenetic data (Taverne 1972; Alves-Gomes and Hopkins 1997; Sullivan et al. 2000; Lavoué et al. 2003). For the two earlier studied taxa names are changed in accordance with recent taxonomic arrangements (Eschmeyer et al. 2020): *Brienomyrus brachyistius* (Gill, 1862) was reported as “*Marcusenius brachistius* Gill” by Uyeno (1973) and *Campylomormyrus rhynchophorus* (Boulenger, 1898) as *C. compressirostris* (Pellegrin, 1924) by Canitz et al. (2016). *Brienomyrus* sp.7 of Ozouf-Costaz et al. (2015) is listed as *Paramormyrops* sp.7 following to Ráb et al. (2016).

**Table 2.** Cytogenetically studied elephantfishes of the family Mormyridae. Diploid chromosome number (2n), karyotypic formula, fundamental number (FN) and geographic origin.

**Taxon**	**2n**	**Karyotypic formula**	**FN**	**Origin**	**References**
**Subfamily Petrocephalinae**					
*Petrocephalus microphthalmus* Pellegrin, 1909	50	2sm + 48a	52	Ogooué Basin, Gabon	Ozouf-Costaz et al. 2015
**Subfamily Mormyrinae**					
*Brevimyrus niger* (Günther, 1866)	48	4m + 2sm + 42a	54	White Nile Basin, Ethiopia	This study
*Brienomyrus brachyistius* (Gill, 1862)	48	1m + 4sm + 2st + 41a	53	Unknown (fish store)	Uyeno 1973
*Campylomormyrus rhynchophorus* (Boulenger, 1898)	48	26m + 4sm + 18a	78	Unknown (laboratory stock)	Canitz et al. 2016
*Cyphomyrus petherici* (Boulenger, 1898)	50	18m + 4sm + 28a	72	White Nile Basin, Ethiopia	This study
*Gnathonemus petersii* (Günther, 1862)	48	10m + 6sm + 32a	64	Unknown (fish store)	Uyeno 1973
	48	18m + 2sm + 28a	68	Unknown (fish store)	Ozouf-Costaz et al. 2015
*Hippopotamyrus pictus* (Marcusen, 1864)	50	24m + 4sm + 22a	78	White Nile Basin, Ethiopia	This study
*Ivindomyrus opdenboschi* Taverne et Géry, 1975	50	10m + 2sm + 38a	62	Ntem River, Gabon	Ozouf-Costaz et al. 2015
*Marcusenius cyprinoides* (Linnaeus, 1758)	50	22m + 4sm + 24a	76	White Nile Basin, Ethiopia	This study
*Marcusenius moorii* (Günther, 1867)	50	4sm + 46a	54	Ntem River, Gabon	Ozouf-Costaz et al. 2015
*Mormyrops anguilloides* (Linnaeus, 1758)	52	52a	52	White Nile Basin, Ethiopia	This study
*Paramormyrops* sp.7	50	2m + 6sm + 42a	58	Ebeigne, Woleu River, Gabon	Ozouf-Costaz et al. 2015
Pollimyrus prope nigricans (Boulenger, 1906)	40	2m + 38a	42	White Nile and Omo-Turkana basins, Ethiopia	Krysanov and Golubtsov 2014
*Stomatorhinus walkeri* (Günther, 1867)	50	2sm + 48a	52	Ogooué Basin, Gabon	Ozouf-Costaz et al. 2015

*Brevimyrus niger* shares the karyotype with 2n = 48 with three other mormyrid taxa, but differs from two of them – *Campylomormyrus rhynchophorus* with FN = 78 and *Gnathonemus petersii* (Günther 1862) with FN = 64 or 68 – by a smaller number of biarmed elements (FN = 54). For third taxon, *Brienomyrus brachyistius*, the unbalanced karyotype with FN = 53 was described in a single specimen (Uyeno 1973). Apart from the unpaired metacentric chromosome of the unclear nature, its karyotype looks similar to that of *Brevimyrus niger*. Both species have two pairs of large biarmed chromosomes, while a pair of uni-armed chromosomes in *Brienomyrus brachyistius* might be substituted by a pair of submetacentrics in *Brevimyrus niger* lineage.

The karyotype with 2n = 50 was found to be dominating in both presently and previously studied mormyrids (three and five taxa, respectively). *Cyphomyrus petherici* (FN = 72), *Hippopotamyrus pictus* (FN = 78) and *Marcusenius cyprinoides* (FN = 76) have more biarmed elements in their compliment than any other mormyrid studied except *Campylomormyrus rhynchophorus* (FN = 78). Congeneric *Marcusenius cyprinoides* and *M. moorii* (Günther, 1867) sharing the same chromosome number differ substantially in their karyotype structure. Up to recently *Cyphomyrus petherici* was considered as belonging to the genus *Pollimyrus* (Taverne 1971; Moritz et al. 2019). Substantial cytogenetic dissimilarity between the single studied species of the latter genus (2n = 40, FN = 42) and *C. petherici* corroborates the change of its generic position (Levin and Golubtsov 2018).

**Figure 2.**
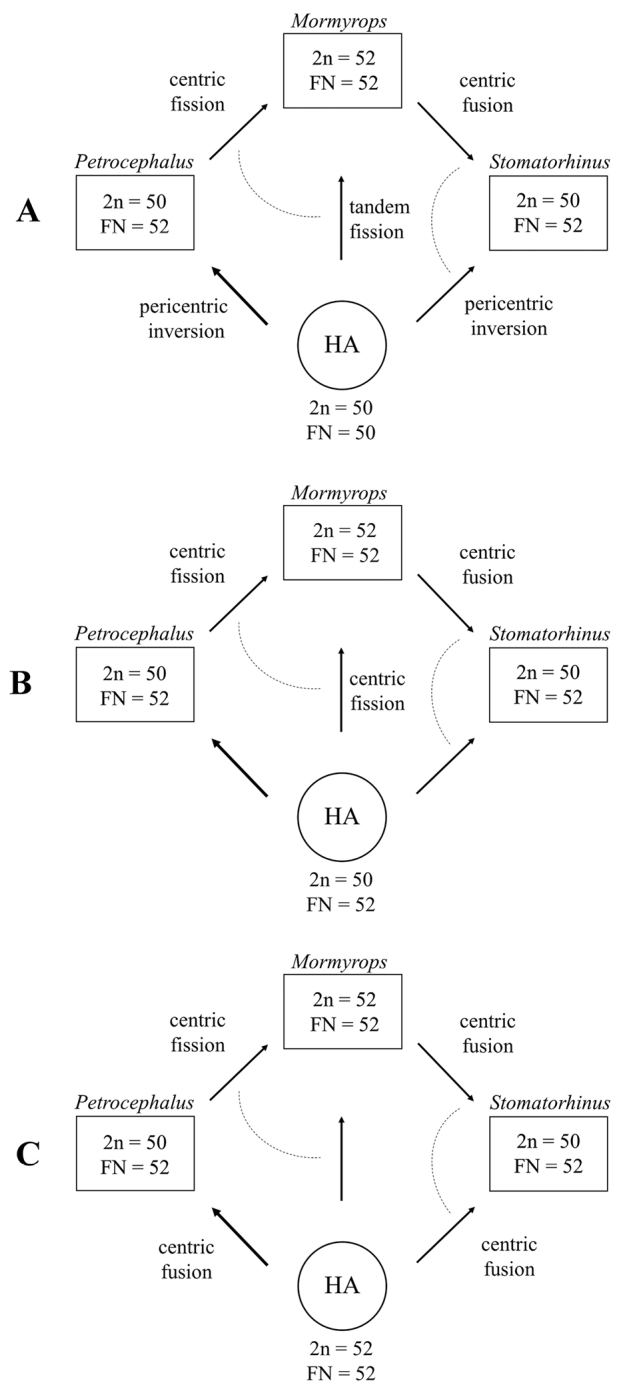
Most parsimonious scenarios of the early karyotype evolution within the family Mormyridae including three variants (**A–C**) of karyotype structure in a hypothetic ancestor (HA) and three studied lineages (the genera *Petrocephalus*, *Stomatorhinus* and *Mormyrops*) with least advanced karyotype structure within the family. The alternative transformations of karyotype structure are joint with a dashed line. The solitary submetacentric pairs in *Petrocephalus* and *Stomatorhinus* are suggested to be not syntenic.

*Mormyrops anguilloides* has a karyotype unique for the mormyrids studied and composed of 52 uni-armed chromosomes. There are two mormyrids – *Petrocephalus microphthalmus* Pellegrin, 1909 and *Stomatorhinus walkeri* (Günther, 1867) – with 2n = 50 and FN = 52. Karyotypes of these three taxa dominated by the uni-armed elements seem to be close to each other and to a hypothetical ancestral karyotype of the family Mormyridae. Mutial trasnformation of these karyotypes could occur in a few evolutionary steps (Fig. 2). It is important that two of the three genera under consideration (*Petrocephalus* and *Mormyrops*) appear to be well-defined basal groups in the family phylogeny (Sullivan et al. 2000; Lavoué et al. 2003). Phylogenetic position of the third genera (*Stomatorhinus*) is unclear. Though it appears in the rather basal position (next to *Petrocephalus*) in the small cladogram by Ozouf-Costaz et al. (2015) based of the mitochondrial cytochrome *b* sequences, in the more extensive mormyrid phylogenies this genus is nested deeper in the phylogenetic trees but in varying and poorly surported positions (Lavoué et al. 2003; Sullivan et al. 2016; Levin and Golubtsov 2018). Unfortunatelly, cytogenetic data for one more genus with the well-defined basal position in the mormyrid phylogeny (*Myomyrus*, stemming out between *Petrocephalus* and *Mormyrops*) are absent.

Based on the simultaneous phylogenetic analysis of molecular data and chromosome number, Canitz et al. (2016) recognized karyotype with n = 24 as the most parsimonius ancestral state for the order Osteoglossiformes, while the haploid chromosome number of n = 24–25 was inferred for the most recent common ancestor of the family Mormyridae. Their analysis, however, did not include the most recent cytogenetic data for several osteoglossomorph clades (Ráb et al. 2016; Barby et al. 2018; Hatanaka et al. 2018; Jegede et al. 2018; Cioffi et al. 2019; de Oliveira et al. 2019). Moreover, the recent genomic data evidence for the ancestral Euteleostomi karyotype of 50 chromosomes with domination by acrocentric elements (Nakatani et al. 2007; Sacerdot et al. 2018; de Oliveira et al. 2019). If the ancestral karyotype of Mormyridae contained 50 uni-armed elements, three chromosomal rearrangements only might produce the observed karyotype structure in the three mormyrid genera (*Petrocephalus*, *Stomatorhinus* and *Mormyrops*) tentatively recognized by us as the least cytogenetically advanced (Fig. 2). The solitary submetacentic pairs in *Petrocephalus* and *Stomatorhinus* are suggested to be not syntenic because of some differences in chromosome morphology (Ozouf-Costaz et al. 2015). If the ancestral karyotype of Mormyridae contained 50 uni-armed elements, it is apparently not retained by any extant mormyrid or osteoglossomorph, in general. Although the karyotype with 2n = 50 is dominating among mormyrids, it contains from 1 to 14 pairs of bi-armed elements (Table 2).

Based on available data the most parsimonius scenarios of the early karyptype evolution in Mormyridae are presented in Figure 2. Three different ancestral karyotypes are considered: 2n = 50 and FN =50 (no bi-armed elements), 2n = 50 and FN = 52 (the only pair of bi-armed elements), 2n = 52 and FN = 52 (no bi-armed elements). The karyotype structure suggested for a hypothetic ancestor could not be retained in any extant mormyrid lineage (Fig. 2A) or retained in *Petrocephalus* (Fig. 2B) or *Mormyrops* (Fig. 2C). It is impossible to judge which of the scenarios considered is more preferable. There are also plenty of less parsimonious scenarios that are not considered by us.

We believe that further cytogenetic studies of various mormyrid taxa may shape the existing views on the karyotype evolution within this diverse group of fish. Looking for the probable interspecific variation of the karyotype structure within the three phylogenetically basal groups (the genera *Petrocephalus*, *Myomyrus*, *Mormyrops*) is of special interest.
